# Enantio- and Diastereoenriched Enzymatic Synthesis of 1,2,3-Polysubstituted Cyclopropanes from (*Z/E*)-Trisubstituted Enol Acetates

**DOI:** 10.21203/rs.3.rs-2802333/v1

**Published:** 2023-04-12

**Authors:** Runze Mao, Daniel J. Wackelin, Cooper S. Jamieson, Torben Rogge, Shilong Gao, Anuvab Das, Doris Mia Taylor, K. N. Houk, Frances H. Arnold

**Affiliations:** 1 Division of Chemistry and Chemical Engineering, California Institute of Technology Pasadena, California, 91125, United States.; 2 Department of Chemistry and Biochemistry, University of California, Los Angeles, California 90095, United States.

**Keywords:** biocatalysis, directed evolution, P450 enzymes, cyclopropanation

## Abstract

In nature and synthetic chemistry, stereoselective [2+1] cyclopropanation is the most prevalent strategy for the synthesis of chiral cyclopropanes, a class of key pharmacophores in pharmaceuticals and bioactive natural products. One of the most extensively studied reactions in the organic chemist’s arsenal, stereoselective [2+1] cyclopropanation, largely relies on the use of stereodefined olefins, which require elaborate laboratory synthesis or tedious separation to ensure high stereoselectivity. Here we report engineered hemoproteins derived from a bacterial cytochrome P450 that catalyze the synthesis of chiral 1,2,3-polysubstituted cyclopropanes, regardless of the stereopurity of the olefin substrates used. Cytochrome P450_BM3_ variant **IC-G3** exclusively converts *(Z)*-enol acetates to enantio- and diastereoenriched cyclopropanes and in our model reaction delivers a leftover *(E)*-enol acetate with 98% stereopurity, using whole *Escherichia coli cells*. **IC-G3** was further engineered with a single mutation to enable the biotransformation of *(E)*-enol acetates to *α*-branched ketones with high levels of enantioselectivity while simultaneously catalyzing the cyclopropanation of *(Z)*-enol acetates with excellent activities and selectivities. We conducted docking studies and molecular dynamics simulations to understand how active-site residues distinguish between the substrate isomers and enable the enzyme to perform these distinct transformations with such high selectivities. Computational studies suggest the observed enantio- and diastereoselectivities are achieved through a stepwise pathway. These biotransformations streamline the synthesis of chiral 1,2,3-polysubstituted cyclopropanes from readily available mixtures of (*Z/E*)-olefins, adding a new dimension to classical cyclopropanation methods.

## Introduction

**Figure 1.**
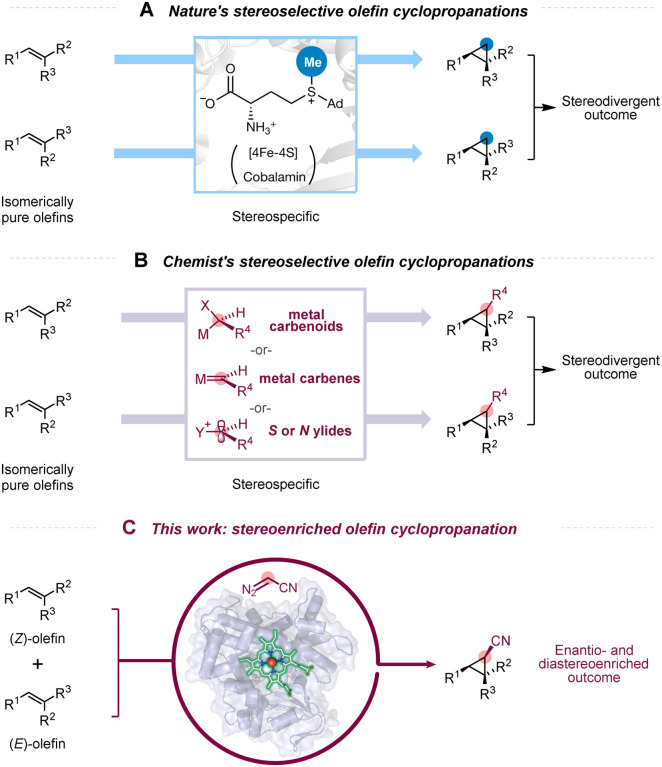
(A) Nature’s stereoselective [2+1] cyclopropanation involving an exogenous C1 unit from SAM via a radical or polar process. This approach is inherently limited to 1,2-substituted cyclopropanes. (B) Stereoselective olefin cyclopropanation methods invented by chemists: [2+1] cyclopropanation of olefins via high-energy intermediates such as metal carbenes, metal carbenoids, or sulfur/nitrogen ylides requires isomerically pure olefins for a stereopure product; (C) This work: enantio- and diastereoenriched olefin cyclopropanation does not require isomerically pure olefins and can form chiral 1,2,3-polysubstituted cyclopropanes. Structural illustrations are adapted from Protein Data Bank (PDB) ID 5UL4 (radical SAM enzyme) and PDB 5UCW (cytochrome P450_BM3_). Ad, adenosyl; R, organic groups; M, metal; X, amino acid; Enz, enzyme.

Chiral 1,2,3-polysubstituted cyclopropanes are prevalent in natural products and bioactive compounds^1–4^ and are versatile building blocks for subsequent downstream manipulation due to their innate ring strain and dense substitution.^5,6^ Stereoselective [2+1] cyclopropanation starting from an olefin and a C1 motif represents a popular disconnection in the retrosynthetic analysis of chiral cyclopropanes; this synthetic approach is widely used in nature as well as in synthetic chemistry (Figure 1a and 1B).^1,7,8^ In nature, *S*-adenosyl methionine (SAM)-dependent methyltransferases transfer exogenous C1 units from the methyl group of SAM to olefins via polar or radical chemistry (Figure 1a).^9^ While highly selective, this approach is inherently limited to 1,2-substituted cyclopropanes, as neither nature nor engineers have succeeded in transferring non-methyl groups with these enzymes. Synthetic chemists, on the other hand, achieve stereoselective [2+1] cyclopropanations via a wider variety of carbon units, such as metal carbenes, metal carbenoids, or sulfur/nitrogen ylides (Figure 1B),^1,7,10–13^ complementing the scope of nature-synthesized cyclopropanes and allowing for access to 1,2,3-polysubstituted cyclopropanes.

Although nature and chemists employ different techniques, stereospecificity is a shared feature of these transformations (Figure 1a and 1B).^1,2,7,9^ When an olefin containing stereochemical information such as a *(Z)*- or *(E)*-configuration is used for [2+1] cyclopropanation, this information is retained in the cyclopropane product. Thus, if an olefin substrate is stereopure, the stereoselectivity of the cyclopropane product can be controlled with relative ease or even predicted *a priori*. On the other hand, if an olefin is a mixture of (*Z/E*)-isomers, the yield and/or selectivity of a desired cyclopropane product will be diminished. Therefore, the geometric purity of the olefin plays a decisive role in the level of stereoinduction and thus determines the stereoselectivity and utility of the method. Because the synthesis of geometrically pure olefins can be challenging,^14,15^ difficult and time-consuming purification of the olefin is often required to ensure high stereopurity of the products.

We were curious whether a catalyst could catalyze conversion of mixtures of (*Z/E*)-olefins into highly enantio- and diastereoenriched cyclopropane products.^16,17^ This would obviate elaborate synthesis or time-consuming separation to construct predefined olefins (Figure 1C). Methods that enable enantio- and diastereoenriched cyclopropanation of (*Z/E*)-olefin mixtures, however, are rare. There are two notable difficulties: (i) The catalyst must recognize the stereochemical information inherent in the olefin substrates. A highly selective catalyst must act exclusively on one substrate while excluding the other. (ii) The catalyst must simultaneously achieve high diastereo- and enantioselectivities, each of which is difficult to achieve. A method that streamlines the synthesis of chiral 1,2,3-polysubstituted cyclopropanes from mixtures of (*Z/E*)-olefins would be of significant synthetic utility.

Enzymes are attractive candidates to meet these challenges, given their ability to exert exquisite control over both the substrates and the stereochemical outcome of chemical reactions.^18^ There are reports highlighting new-to-nature transformations,^19^ including olefin cyclopropanation, achieved by expanding the already enormous catalytic repertoire of the (iron)-heme-containing cytochrome P450 family.^20,21^ Cytochromes P450 are excellent candidates for discovery of non-natural activities due to their structural flexibility and remarkable promiscuity.^22^ A decade ago, we described repurposing a cytochrome P450 for non-natural cyclopropanation of styrenes with diazoesters, yielding cyclopropanes with high levels of selectivity via a putative carbene transfer process.^21^ Since that first report, a plethora of hemoprotein-catalyzed carbene and nitrene transfer reactions have been developed.^20,23,24^ The activity and selectivity of these biocatalytic transformations are often complementary to the state-of-the-art processes based on small-molecule catalysts, making them a valuable addition to the synthetic chemist’s toolbox. Notably, reports of biocatalytic cyclopropanation have largely been limited to terminal olefins and stereodefined internal olefins, sidestepping the issue of using geometrically different olefins (Figure 1C).^21,25–31^ Based on these precedents, we saw opportunities to leverage cytochromes P450 for stereoenriched cyclopropanations from (*Z/E*)-mixed internal olefins.

## Results and discussion

### Initial screening and directed evolution of alkyl transferase IC-G3.

We commenced our study by focusing on the cyclopropanation reaction between a 1:1 *Z/E* mixture of butyrophenone-derived enol acetate **1a** and diazoacetonitrile **2a.** These reagents were chosen for several reasons: (i) enol acetates will be transformed into cyclopropyl acetates, which are synthetically and pharmaceutically relevant compounds;^32,33^ (ii) the opposite polarities of the different groups on the alkene (polar acetyl and nonpolar phenyl) can help the enzyme distinguish different stereoisomers; (iii) diazoacetonitrile **2a** was chosen for its strong electron-withdrawing character, small steric profile, and the valuable nature of the nitrile group. The nitrile substituent enhances the electrophilicity of the carbenoid and promotes reactions with the nucleophilic enol acetates. Additionally, the small steric profile of the nitrile is less likely to impede accommodation of diverse, sterically hindered substrates in the enzyme active site. Moreover, nitrile groups are valuable moieties that can be readily transformed into a variety of functional groups.

**Figure 2.**
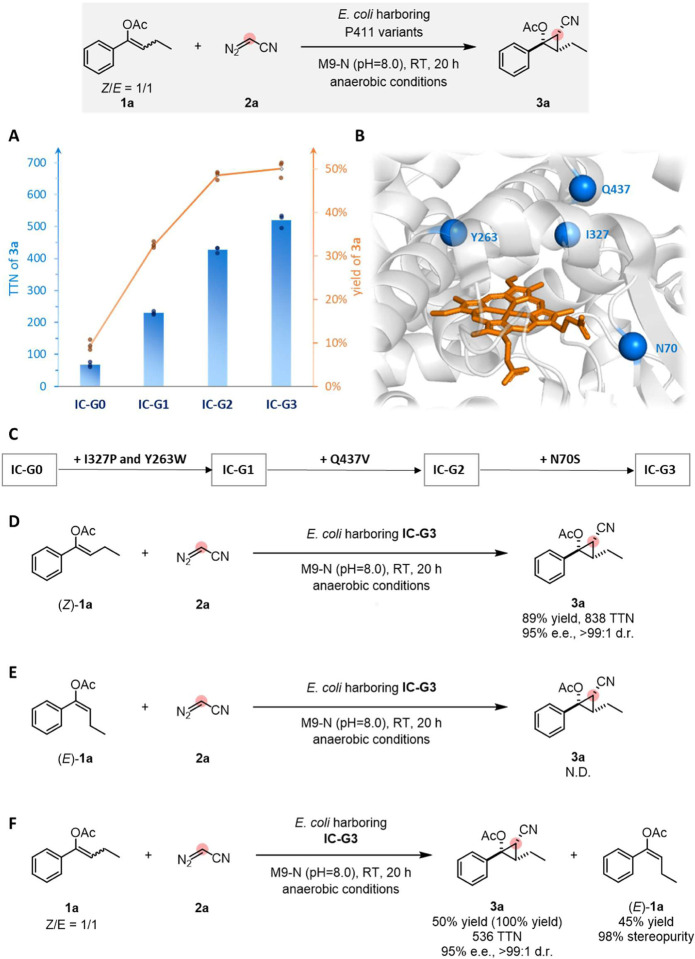
Directed evolution for enantio- and diastereoenriched cyclopropanation. Reaction conditions: 3 mM **1a**, 37 mM **2a**, *E. coli* whole cells harboring P411 variants (OD_600_ = 30) M9-N aqueous buffer (pH 8.0); 10% v/v EtOH (co-solvent); room temperature; anaerobic conditions; 20 h. (A) Directed evolution of enantio- and diastereoenriched alkyltransferase **IC-G3**. Reactions were performed in triplicate (n = 3). Yields and TTNs reported are the means of three independent experiments; (B) The mutated residues (N70, Y263, I327, and Q437) conferring activity increases are highlighted in the enzyme active site structure (of closely related P411 variant **E10** (PDB ID: 5UCW)); (C) Evolutionary trajectory for enantio- and diastereoenriched cyclopropanation; (D) **IC-G3**-catalyzed cyclopropanation reaction using *(Z)*-**1a**; *(E)* **IC-G3**-catalyzed cyclopropanation reaction using *(E)*-**1a**; (F) **IC-G3**-catalyzed cyclopropanation reaction using a mixture of (*Z/E*)-**1a**.

We screened a panel of 48 hemoproteins previously engineered for different carbene and nitrene transformations, including variants of cytochromes P411 (cytochromes P450 with an axial serine ligand), in intact *Escherichia coli* (*E. coli*) cells. Compounds **1a** and **2a** were combined with the heme proteins expressed in whole *E. coli* cells at room temperature under anaerobic conditions. The resulting reaction mixtures were analyzed after 20 hours for the formation of cyclopropane product **3a**. A truncated P411 variant lacking the FAD domain (named **IC-G0** in this new lineage) that was previously used for intramolecular nitrene insertion into C(sp^3^)–H bonds^34^ showed the best activity in this initial screen, providing a total turnover number (TTN) of 68 and 9% yield (Figure 2a; see Supplementary Table1 for details). The heme domain of this variant has seven mutations (A74G, V78L, L263Y, T327I, T436L, L437Q, and S438T) with respect to the previously reported “**E10**” variant of P450_BM3_, which has a solved crystal structure (Figure 2B, PDB ID: 5UCW).^35^ Control experiments showed that formation of **3a** is neither catalyzed by the heme cofactor alone, nor is it produced by the cellular background (see Supplementary Table 1 for details).

Since **IC-G0** showed promising activity toward the desired cyclopropanation reaction, we decided to revisit other enzymes in the collection that are closely related to **IC-G0**.^34,36^ To our delight, variant **IC-G1**, with mutations I327P and Y263W relative to **IC-G0**, showed higher activity than **IC-G0** (**IC-G1** can catalyze the formation of **3a** in 33% yield and 230 TTN, Figure 2a). We chose variant **IC-G1** as the parent for directed evolution via iterative rounds of site-saturation mutagenesis (SSM) and screening, targeting amino acid residues close to the heme cofactor (Figure 2B). Mutation Q437V, which resides on the flexible loop directly above the heme (Figure 2B), increased the TTN to 427 (49% yield of **3a**, Figure 2B). Exploring more amino acid residues in the enzyme pocket using SSM identified the N70S mutation and increased the TTN to 536 (50% yield, 95% e.e. and >99:1 d.r. of **3a**). We found it difficult to push the yield higher at this stage. Given the 1:1 ratio of (*Z/E*)-**1a**, we speculated this 50% yield ceiling meant the enzyme was reacting with only one isomer of the starting material.

To verify this and gain more insight into the biotransformation, stereoisomers *(Z)*-**1a** and *(E)*-**1a** were used individually as the substrate for **IC-G3**-catalyzed cyclopropanation reactions with diazoacetonitrile **2a** under standard conditions (Figure 2D and 2E). **IC-G3** converts pure *(Z)*-**1a** into **3a** with 89% yield and 838 TTN (95% e.e. and >99:1 d.r.) but does not react with pure *(E)*-**1a** to form a cyclopropane product (Figure 2D and 2E). Encouraged by this, we re-examined the reactions with a 1:1 *Z/E* mixture of **1a** and found the remaining starting material was highly enriched in *(E)*-isomer (*Z/E* = 2:98; Figure 2F). This is interesting, as pure *(E)*-olefins are more difficult to obtain due to their lower thermostabilities than *(Z)*-olefins, and only a handful of methods are reliable for the diastereoselective synthesis of these *(E)*-olefins.^37^ In this regard, in addition to the generation of high-value added chiral 1,2,3-polysubstituted cyclopropanes, **IC-G3** also holds potential utility in delivering stereopure *(E)*-enol acetates.

## Substrate scope study

**Figure 3.**
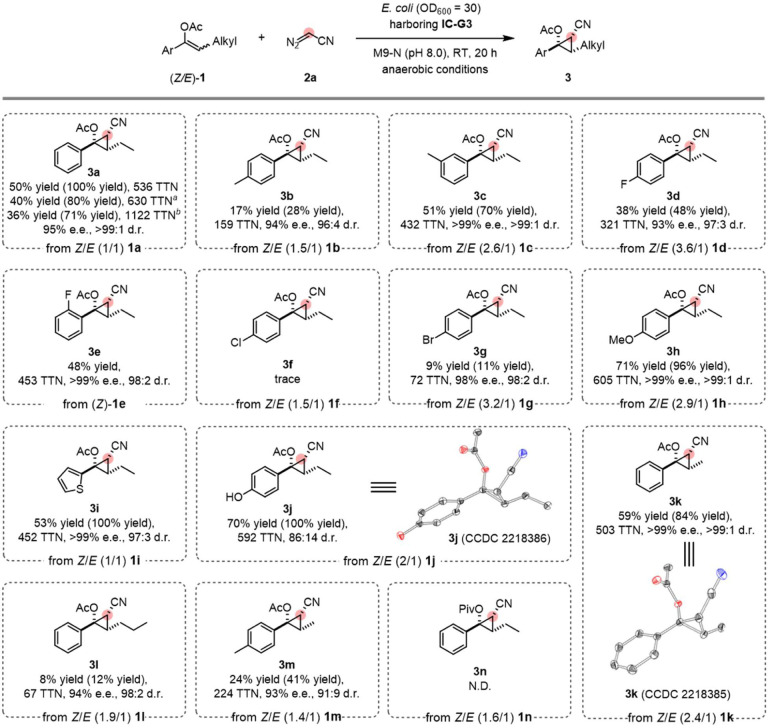
Substrate scope study. Reaction conditions: 3 mM **1**, 37 mM **2a**, *E. coli* whole cells harboring **IC-G3** (OD_600_ = 30) M9-N aqueous buffer (pH 8.0); 10% v/v EtOH (co-solvent); room temperature; anaerobic conditions; 20 h. Ar, aryl groups; Alkyl, alkyl groups. Yields are based on mixed enol acetates. Yields in parenthesis are based on *(Z)*-isomer only. ^a^5 mM 1 and 35 mM **2a** were used; ^b^10 mM **1** and 30 mM **2a** were used; N.D. = no product was detected.

We surveyed the activity of **IC-G3** on a series of trisubstituted enolate substrates under the standard whole-cell reaction conditions (Figure 3). **IC-G3** catalyzes cyclopropanation of *Z/E* mixtures of diverse *α*-aryl, *β*-alkyl-substituted enolates **1** with diazoacetonitrile **2a**, delivering the desired products **3** in synthetically useful yields and excellent diastereo- and enantioselectivities (up to >99:1 d.r. and >99% e.e., Figure 3), regardless of the stereopurity of the olefin substrates used. Substrates with diverse substituents on the *α-*aryl group are compatible with this reaction. Electron-donating, -neutral, and -withdrawing substituents on the aromatic rings were all compatible (**3a–3n**; Figure 3), affording 1,2,3-polysubstituted cyclopropanes with uniformly high levels of diastereo- and enantioselectivities. *Para*- and *meta*-substituted *α*-aryl enolates both reacted well with diazoacetonitrile **2a** to give the corresponding cyclopropanes (3b–3c, Figure 3). Substrates bearing a halogen functional group, such as fluoro- (**1d** and **1e**), and bromo- (**1g**), were tolerated to generate cyclopropanated products (**3d–3g**, Figure 3) with excellent selectivities. A chloro group (**1f**, Figure 3), however, is detrimental to the cyclopropanation reaction, giving a trace amount cyclopropane product **3f** (Figure 3). Introducing an electron-donating group can increase the yield of cyclopropanes (**3h** and **3j**, Figure 3). Notably, a phenolic hydroxyl group, which is generally incompatible with small-molecule carbene transfer reactions due to its nucleophilicity,^37^ is well tolerated by the enzymatic system (3j, Figure 3), which highlights the catalyst’s functional group tolerance. Structural perturbations, such as the substitution of the aryl ring by thiophene (3i, Figure 3), are also well accepted. Furthermore, *β*-substituents bearing various alkyl chains (1k–1m, Figure 3) could be transformed to the corresponding cyclopropyl acetates with high levels of diastereo- and enantioselectivities (up to >99:1 d.r. and >99% e.e.). We also attempted to replace the enol acetyl group with a pivaloyl group, but no cyclopropanated product was observed (**3n**, Figure 3), likely because the bulky pivaloyl group prevented recognition of **1n** by the enzyme.

To showcase the utility of **IC-G3**, we challenged the biocatalyst with higher substrate loadings. Under standard conditions and an alkene concentration of 3 mM 1:1 *Z/E*-**1a**, the TTN of the template reaction was 536 (**3a**, Figure 3). Encouragingly, increased substrate loadings also resulted in synthetically useful yields, while increasing the TTNs to 630 (5 mM) and 1122 (10 mM) (**3a**, Figure 3).

## Engineering diastereomer-differentiating carbene transferase IC-G4

**Figure 4.**
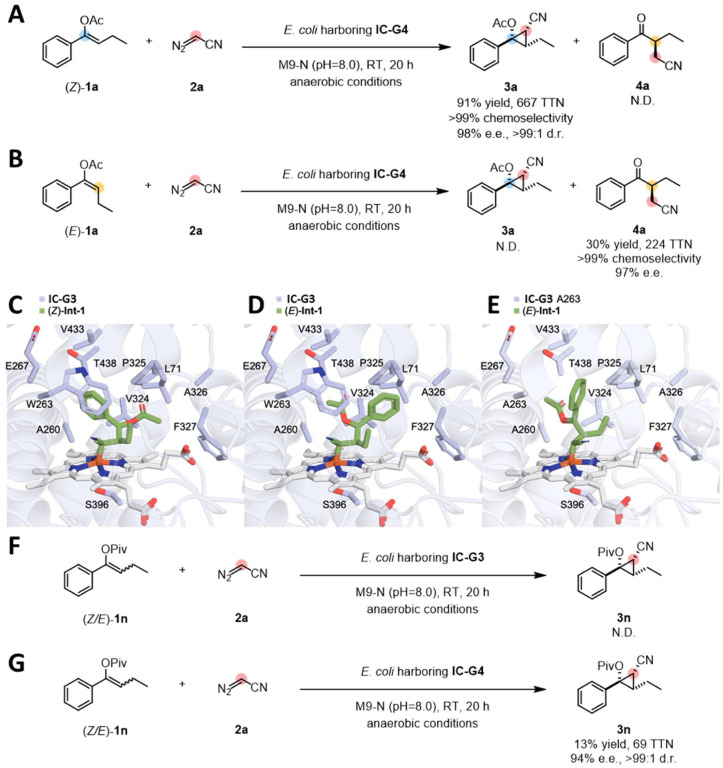
Discovery of the diastereomer-differentiating alkyl transferase IC-G4 and its activity toward divergent alkyl transfer reactions. (A) **IC-G4**-catalyzed alkyl transfer reaction using *(Z)*-**1a**; (B) **IC-G4**-catalyzed alkyl transfer reaction using *(E)*-**1a**; Reaction conditions: 3 mM *(Z)*-**1a** or *(E)*-**1a**, 37 mM **2a**, *E. coli* whole cells harboring **IC-G4** (OD_600_ = 30) M9-N aqueous buffer (pH 8.0); 10% v/v EtOH (co-solvent); room temperature; anaerobic conditions; 20 h; Docking simulations of: (C), *(Z)*-**Int-1** with **IC-G3**; (D), *(E)*-**Int-1** with **IC-G3**; *(E)*, *(E)*-**Int-1** with **IC-G3** A263; (F) **IC-G3**-catalyzed alkyl transfer reaction using a mixture of *Z/E* (1.6/1)-**1n**; (G) **IC-G4**-catalyzed alkyl transfer reaction using a mixture of *Z/E* (1.6/1)-**1n**; Reaction conditions: 3 mM (*Z/E*)-**1n**, 37 mM **2a**, *E. coli* whole cells harboring **IC-G3** or **IC-G4**(OD_600_ = 30); yields were calculated based on all **1n** used.

Re-examining site-saturation mutagenesis libraries based on **IC-G3**, we found that mutations at residue 263 resulted in a significant reduction in remaining starting material and the appearance of a new product peak in some variants. Mutation of the tryptophan to smaller, more flexible methionine yielded the largest new product peak, which was identified as *α*-branched ketone product **4a**. The specificity of this new variant, **IC-G4**, was tested with pure *(Z)*-**1a** and *(E)*-**1a** separately (Figure 4a and 4B). Interestingly, **IC-G4** not only converts *(Z)*-**1a** and *(E)*-**1a**, it converts them into different products. To be specific, *(Z)*-**1a** was converted to the cyclopropane product **3a** in 91% yield with high selectivities (98% e.e. and >99:1 d.r.; Figure 4a), but no **4a** was detected (>99% chemoselectivity of **3a** over **4a**; Figure 4a). Conversely, **IC-G4** catalyzes the transformation of *(E)*-**1a** into **4a** in 30% yield and with 97% e.e., but **3a** was not detected (>99% chemoselectivity of **4a** over **3a**; Figure 4B). We also investigated the substrate scope of **IC-G4-**catalyzed diastereomer-differentiating transformations (see Supplementary Figure 1). **IC-G4** accepts a variety of *Z/E* mixtures of *α*-aryl, *β*-alkyl-substituted enolates **1** with diazoacetonitrile **2a**, delivering the desired cyclopropane products **3** (up to quantitative yield (yields calculated based on the *(Z)*-isomer), >99% chemoselectivity, >99:1 d.r. and >99% e.e., Supplementary Figure 1) and *α*-branched ketone products **4** (Figure 3 Supplementary Figure 1) in synthetically useful yields and excellent diastereo- and enantioselectivities.

**Figure 5.**
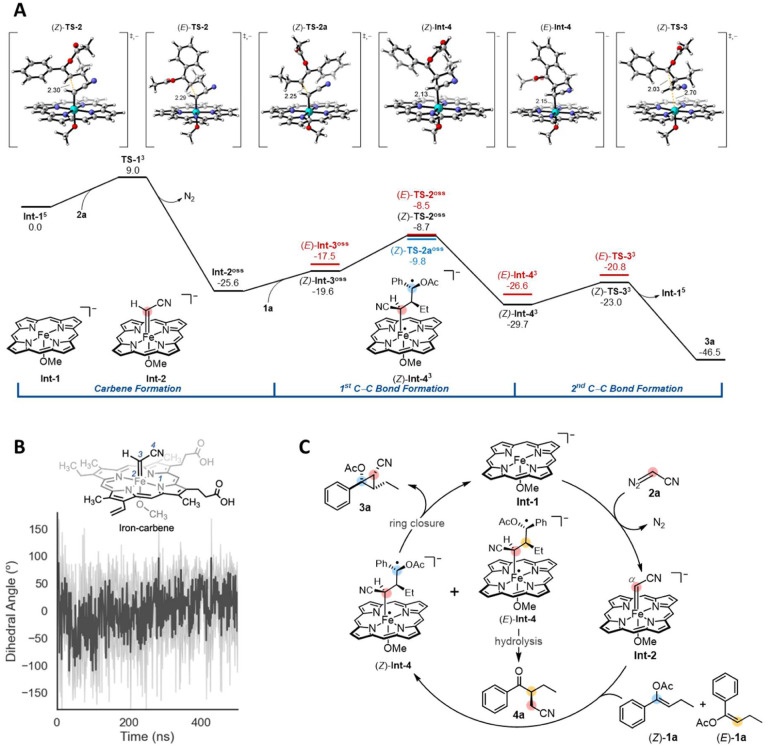
Proposed stepwise pathway for enantio- and diastereoenriched enzymatic cyclopropanation. (A) Calculated energy diagram for cyclopropanation (standard heme model at the B3LYP-D3(BJ)-CPCM(Et_2_O)/def2-TZVP//B3LYP-D3(BJ)/def2-SVP level of theory; energies are Gibbs free energies in kcal mol^−1^; superscripts correspond to the spin state; distances are given in Å); (B) Molecular dynamics simulations on the iron-carbene intermediate; (C) A proposed catalytic cycle of the biocatalytic cyclopropanation.

As **IC-G3** and **IC-G4** differ only by a single amino acid, at residue 263, we were curious to understand this site’s role in substrate recognition. We first conducted docking simulations to understand how **IC-G3** catalyzes the cyclopropanation of *(Z)*-**1a** to form **3a** and not **4a**. To do this, we turned to quantum mechanics to calculate the reaction pathway and docked in the heme-coordinated intermediate (*(Z)*-**Int-1** in Figure 5A) that forms after nucleophilic attack by the *β*-carbon of *(Z)*-**1a** with the electrophilic heme-carbene. Docking simulations predict that *(Z)*-**Int-1** fits tightly in the active site of **IC-G3** (Figure 4C). The phenyl and acetyl handles of *(Z)*-**Int-1** form stabilizing hydrophobic interactions with the side chains of residues V324 and W263. The conformation of docked *(Z)*-**Int-1** is distorted toward a geometry that allows for direct ring closure to the observed cyclopropane **3a** product. In the structure (Figure 4C), there is a polarity mismatch: the phenyl group is situated adjacent to the hydrophilic side chains of T438 and E267 and the acetyl group is forced into a hydrophobic pocket surrounded by V324 and P325. By docking **Int-1** with free rotation around all single bonds, we discovered a second pose *(E)*-**Int-1** that must form from *(E)*-**1a** based on the orientation of the phenyl and acetyl groups (Figure 4D). In the *(E)*-**Int-1** pose, the phenyl group is wedged between W263 and P325 forming stabilizing hydrophobic interactions, and the acetyl group is pointed toward the hydrophilic active site region near T438 – here, the substituents have rotated to a conformation that allows the polarity of the substituents to match the active site residue polarity. Arguably, this *(E)*-**Int-1** pose is an artifact of the docking simulation that forces other probable poses when there is none. This is corroborated by the experimental evidence that no products derived from *(E)*-**1a** are observed when **IC-G3** is the catalyst. In silico mutation of W263 to alanine (Figure 4E) significantly enlarges the active site and allows rotation of the phenyl group into a conformation < 1.7 Å from where W263 would have been. Furthermore, rotation of the phenyl group effectively removes stabilization of the benzylic position and pushes this partial charge onto the oxygen, further facilitating the loss of the acetyl group and formation of the *α*-alkyl transfer product **4a** (Figure 4B and 4D). Perhaps from this *(E)*-**Int-1** conformation – when the acetyl group is near hydrophilic T438 – hydrolysis and loss of an acetic acid precludes cyclopropane formation and leads to *α*-alkyl transfer product **4a** instead (Figure 4B and 4D). Similarly, introducing the W263M mutation (**IC-G4**) shows a docking position similar to the alanine mutant, implying the exclusion of *(E)*-**1a** in **IC-G3** is due to the smaller active site cavity compared to the other variants. This is corroborated by the fact that using a bulkier pivaloyl protecting group on the oxygen shuts down cyclopropanation with **IC-G3** (**3n**, Figure 3 and 4F), whereas **IC-G4** was able to catalyze the transformation of (*Z/E*)-**1n** and **1f** to **3n** and **3f** in 13% (69 TTN) and 39% (170 TTN) yields, respectively, with excellent selectivities (Figure 4G, and Supplementary Figure 1). We attribute these differences to the enlarged active site of **IC-G4**.

## Computational modeling supports a stepwise pathway

We were curious how this enzyme can overcome potential severe steric constraints in order to cyclopropanate highly substituted olefins. Classical cyclopropanation reactions following a concerted reaction pathway encounter steric clashes which often make reactions with densely functionalized olefins challenging.^25,38^ The potential energy surface for cyclopropanation was calculated by means of density functional theory (DFT) calculations using a standard heme model at the B3LYP-D3(BJ)-CPCM(Et_2_O)/def2-TZVP//B3LYP-D3(BJ)/def2-SVP level of theory (Figure 5A).^39–44^ These model calculations indicate that the key cyclopropanation step takes place via a stepwise mechanism involving two distinct C–C bond formations on an open-shell singlet (OSS) and a triplet surface instead of a concerted process. Upon rapid formation of the iron-carbene **Int-2** via transition state **TS-1** and subsequent coordination of **1a**, the nucleophilic *β*-carbon of the enol acetate attacks the electrophilic carbene via *(E)-***TS-2** and *(Z)*-**TS-2** to form two conformations of the intermediate, *(E)*-**Int-4** and *(Z)*-**Int-4**, respectively (Figure 5A and 5C). This first bond-forming event with both *(E)*-**1a** and *(Z)*-**1a** results in the formation of the (S)-configured stereocenter (Figure 5A and 5C). Transition states *(E)*-**TS-2** and *(Z)*-**TS-2** are nearly isoenergetic (ΔΔG^‡^ = 0.2 kcal·mol^–1^) which indicates an almost equal rate of formation of intermediates *(E)*-**Int-4** and *(Z)*-**Int-4** (Figure 5A). Calculations revealed an enantiomeric transition state, *(Z)*-**TS-2a**, which is preferable to *(Z)*-**TS-2** by 1.1 kcal·mol^–1^. This result indicates that the enzyme active site is configured in such a way to disfavor binding and formation of *(Z)*-**TS-2a**. We hypothesize that this is due to the conformation of the iron-carbene intermediate in the enzyme active site. Molecular dynamics (MD) simulations on this iron-carbene intermediate indicate that the N–Fe–C–C dihedral has an average angle of 0° and the highest probabilities were found at +50° and −60° (Figure 5B). These results suggest that formation of *(Z)*-**TS-2a** in the enzyme requires *(Z)*-**1a** to orient both ethyl and acetoxy substituents towards the sterically more congested back of the active site, thereby likely resulting in a destabilization of *(Z)*-**TS**-**2a** compared to *(Z)*-**TS-2**. Afterwards, ring-closing C–C bond formation from intermediates *(E)*-**Int-4** and *(Z)*-**Int-4** takes place via transition states *(Z)*-**TS-3** and *(E)*-**TS-3**, respectively. *(Z)*-**TS-3** leads to the observed cyclopropane product **3a**, whereas the activation free energy for *(E)*-**TS-3** is 2.2 kcal·mol^–1^ higher, indicating a 100-fold more difficult reaction. This energetic value is corroborated by the experimental result that only the cyclopropane **3a** is observed. Presumably, due to this larger energetic barrier, *(E)*-**Int-4** undergoes rapid hydrolysis to form the *α*-alkyl transfer product **4a**. Based on this computational evidence, this process seems more likely than formation of the cyclopropane via *(E)*-**TS-3** and concomitant ring opening to form **4a**. However, further studies are required to validate this proposal. A catalytic cycle based on these computational results is proposed in Figure 5C.

## Summary and conclusion

In summary, we have developed a novel biocatalytic platform for highly enantio- and diastereoselective cyclopropanation of mixed (*Z/E*)-trisubstituted enol acetates. These biocatalysts are fully genetically encoded, allowing for rapid tuning and reconfiguration via manipulation of the DNA sequence. Through directed evolution, we discovered two P411 variants, **IC-G3** and **IC-G4**, where **IC-G3** exclusively catalyzes cyclopropanation and **IC-G4** enables diastereomer-differentiating transformations, both with excellent selectivities. DFT calculations suggest a stepwise mechanism for these biotransformations, and our docking simulations highlight the critical role of site 263 in controlling the active-site accommodation of the (*Z/E*)-olefinic isomers. Our approach differs from traditional carbene-transfer cyclopropanations by converting hard-to-isolate olefinic mixtures into a single chiral cyclopropane product with exceptional selectivities. We anticipate that this biocatalytic platform will expedite the synthesis of chiral 1,2,3-polysubstituted cyclopropanes from readily available olefinic isomers.
